# Evaluation of new expert rules for the detection and differentiation of carbapenemase-producing Enterobacterales within the French epidemiological context

**DOI:** 10.1128/jcm.00222-26

**Published:** 2026-05-28

**Authors:** Cécile Emeraud, Lélia Abad, Virginie Crolet, Sophie Dos Santos, Gaëlle Noel, Sarah Rivat, Gilles Zambardi, Laurent Dortet

**Affiliations:** 1 Team "Resist" UMR1184 "Immunology of Viral, Auto-Immune, Hematological and Bacterial diseases (IMVA-HB)", Faculty of Medicine, INSERM, Université Paris-Saclay, CEA, LabEx LERMIT 537860 , Le Kremlin-Bicêtre, France; 2 Bacteriology-Hygiene Unit, Bicêtre Hospital, Assistance Publique-Hôpitaux de Paris 41664 , Le Kremlin-Bicêtre, France; 3 Associated French National Reference Center for Antibiotic Resistance: Carbapenemase-Producing Enterobacteriaceae 26930, Le Kremlin-Bicêtre, France; 4 SEPSIS Comprehensive Center- IHU SEPSIS, Le Kremlin-Bicêtre, France; 5 bioMerieux SA 1896 , Marcy l’Etoile, France; Mayo Clinic, Rochester, Minnesota, USA

**Keywords:** carbapenemase-producing Enterobacterales, OXA-48-like, OXA-244, automated AST, disk diffusion, CA-SFM, VITEK2

## Abstract

The increasing prevalence of carbapenemase-producing Enterobacterales (CPE) requires reliable diagnostic tools. This study evaluated the performance of the VITEK2 Advanced Expert System, a software interpretation program combined with VITEK2 AST results, for CPE detection, and the ability of BIOART expert rules to identify carbapenemase types. Antimicrobial susceptibility testing was performed using VITEK2 (N436 + XN28 cards) and disk diffusion on a collection of 244 isolates representative of French epidemiology, including 145 CPE, 51 non-CPE carbapenem-resistant Enterobacterales (CRE), and 48 non-CRE. A dedicated OXA-244/484 rule was assessed on a collection of 36 OXA-244/484 producers, known to be difficult to detect. The VITEK2 N436/AES and CA-SFM algorithm demonstrated similar sensitivities (93.8% vs 97.2%) and specificities (53.5% vs 49.5%) for CPE detection. Most detection errors involved OXA-244/484 producers. Application of the dedicated OXA-244/484 rule enabled 100% detection. BIOART rules identified the single carbapenemase class with no other mechanism reported in 83 cases, the correct class with additional mechanisms reported in 30 cases, and no carbapenemase class, or an incorrect carbapenemase class were assigned in 6 and 5 cases, respectively. Twelve isolates were outside the scope of BIOART rule (IMI or multiple carbapenemases). Single correct carbapenemase class assignment was achieved in 97.5% of NDM producers, 54.5% of OXA-48-like producers, and 57.1% of VIM producers. All KPC producers were correctly identified as KPC producers, although carbapenem impermeability was systematically co-reported, preventing single unambiguous assignment. The integrated VITEK2 AES/BIOART solution provides an efficient approach for CPE screening and classification, particularly for NDM and KPC-producers.

**IMPORTANCE:**

Rapid and reliable detection of carbapenemase-producing Enterobacterales (CPE) is essential for infection control, epidemiological surveillance, and optimized antimicrobial therapy. As the prevalence and diversity of carbapenemases increase, diagnostic tools capable not only of detecting CPE but also predicting carbapenemase classes are increasingly needed to guide treatment decisions. In Europe, the emergence of OXA-48-like variants such as OXA-244 and OXA-484, which exhibit low hydrolytic activity to carbapenems and temocillin, complicates routine phenotypic detection and may lead to diagnostic failure. This study evaluates an automated solution integrating the VITEK2 Advanced Expert System and BIOART expert rules for CPE detection and classification in a representative French epidemiological context. We demonstrate that this integrated approach provides accurate and rapid screening, with good performance for carbapenemase class prediction, particularly for NDM and KPC producers. The implementation of a dedicated rule significantly improves detection of difficult-to-identify OXA-244/OXA-484 producers, addressing an important diagnostic gap.

## INTRODUCTION

In recent years, an increasing number of carbapenemase-producing Enterobacterales (CPE) have been reported (1, 2). Due to the extremely limited therapeutic options available, the emergence and spread of CPE infections represent a major public health concern (3–5). In addition to their resistance to carbapenems, CPE commonly exhibit resistance to β-lactams including extended-spectrum cephalosporins (e.g., cefotaxime, ceftriaxone, ceftazidime, or cefepime), and to other antibiotic classes (6). Detection of carbapenemases is crucial for epidemiological surveillance, infection control, and patient management, as well as to guide the use of novel antimicrobial agents specifically designed to target certain carbapenemase mechanisms (7, 8).

Since 2021, an increasing number of OXA-244/OXA-484, OXA-48-like variants with weaker hydrolytic activity toward carbapenems and temocillin, have been reported in Europe (9, 10). The low-level carbapenemase activity of these variants contributes to significant detection challenges in routine diagnostic workflows. In France, OXA-244 enzymes are estimated to account for approximately 16% of the OXA-48-like variants (11). In 2022, to improve the detection of OXA-244/-484 producers but also of VIM producers, the Antibiogram Committee of the French Microbiology Society (CA-SFM) introduced a refined algorithm based on disk diffusion (DD) (12). This DD-based approach demonstrated high sensitivity (97.8%) with positive and negative predictive values of 89.7% and 80.9%, respectively (13). However, upon positive screening, confirmatory tests such as lateral flow immunochromatographic assay or molecular biology remain mandatory for enzyme classification (12). Although these methods offer high sensitivity, they are associated with substantial costs, which may limit their routine use in certain settings.

BioMérieux’s VITEK2 Advanced Expert System (AES) automatically identifies resistance phenotypes (e.g., carbapenemase production or reduced permeability to carbapenems) based on approximated MIC results generated by the VITEK2 system (MIC_VITEK_). This expert system is widely used in France and in several other European countries, although its implementation may vary depending on national guidelines and local laboratory practices. While the AES can indicate the presence of a carbapenemase, it does not specify its type. To address this limitation, the CARBA BIOART rules were recently developed to complement the AES by enabling carbapenemase class determination (OXA-48-like, KPC, and MBL). These rules aim to improve the interpretability of AST reports and support a more targeted antibiotic therapy in alignment with recent recommendations, with the goals to optimize the use of antibiotics, limit the emergence of resistance, and maintain patient safety (5, 7, 14). A recent US-based study demonstrated promising sensitivities for these rules in detecting MBL (97%), KPC (76%), and OXA-48-like (90%) producers (15). However, only a limited number of OXA-48-like producers (*n* = 19) were included in that study, reflecting their low prevalence in the United States, which limits the generalizability of these findings to other epidemiological contexts. Moreover, the limited number of non-carbapenemase-producing carbapenem-resistant Enterobacterales (non-CPE CRE) did not allow for a reliable assessment of possible false positives.

This study aimed to compare the performance of the VITEK2 AES/BIOART solution to the CA-SFM algorithm for CPE screening within the French epidemiological context.

## MATERIALS AND METHODS

### Strain collection and molecular characterization

All isolates were obtained from the French National Reference Center (F-NRC) for Antibiotic Resistance. The main analysis was performed on a collection of 244 clinical isolates, including 145 CPE, 51 non-CPE CRE (with reduced susceptibility to at least one carbapenem (ertapenem, imipenem, or meropenem)), and 48 non-CRE, collected between April and December 2023. CPE isolates were selected to reflect the current epidemiological distribution of carbapenemase types in France during this period, in order to ensure representativeness of each carbapenemase class and minimize overrepresentation of closely related clones. A second subset of 33 OXA-244- and 3 OXA-484-producing isolates was collected between April and June 2024 to test the ability to detect and identify these difficult-to-detect variants. Bacterial species identification was performed using MALDI-TOF (Bruker). All isolates underwent antimicrobial susceptibility testing by disk diffusion. The presence of ESBL and/or high-level AmpC production in non-CRE isolates was inferred from antimicrobial susceptibility testing profiles according to EUCAST interpretive criteria (EUCAST, 2023, https://www.eucast.org/). In case of suspected high-level AmpC production, testing was performed using cloxacillin-supplemented Mueller-Hinton agar.

All CPE and non-CPE CRE isolates were fully characterized by whole-genome sequencing (Illumina technology), as previously described (16) (BioProject PRJNA1448025). Resistance genes were identified using ResFinder v4.7.2, and multilocus sequence typing (MLST) was performed using the PubMLST database. Non-CRE isolates were not sequenced; the presence of ESBL and/or high-level AmpC production was inferred from antimicrobial susceptibility testing profiles, thus the underlying β-lactamase genes were not characterized. Classification as non-CRE was based on extensive phenotypic expertise from F-NRC, based on MIC/inhibition zone of different antibiotic markers. In routine practice, in case of any doubt, additional confirmatory testing (e.g., NG-Test CARBA 5, and cloxacillin-supplemented agar) is systematically performed.

### Carbapenemase detection and classification using VITEK2 AES/BIOART solution

All isolates were sub-cultured on CHROMID CPS Elite agar (bioMérieux, Marcy l’Etoile, France) and incubated for 18–24 h at 35°C, followed by antimicrobial susceptibility testing on the VITEK2 N436 and VITEK2 XN28 cards (bioMérieux). Card compositions are detailed in Table S4. The VITEK2 AES (v9.03.4) interpreted the phenotypes based on MIC results obtained from one or both cards. When both cards were used, the CARBA BIOART rules were activated to determine the carbapenemase type (i.e., possibility of OXA-48-like, KPC, and MBL). These rules were applied to the following species*: Escherichia coli*, *Klebsiella* spp., *Enterobacter cloacae* complex, *Serratia marcescens*, and *Citrobacter freundii*. Depending on the species, carbapenemase-type predictions were based on MIC values for piperacillin-tazobactam, cefotaxime, ceftazidime, ceftriaxone, cefepime, aztreonam, ertapenem, meropenem, temocillin, meropenem-vaborbactam, imipenem-relebactam, ceftazidime-avibactam, and ceftolozane-tazobactam. In addition, the meropenem/meropenem-vaborbactam MIC ratio was used to support the interpretation of carbapenemase type.

Following the first set of results, a specific rule targeting OXA-244/OXA-484 variants was developed for *E. coli* isolates with an ertapenem MIC_VITEK_ = 0.25 mg/L (available on the first tested N436 urinary card).

The BIOART rules are available to clinical laboratories; however, they are not CE-IVDR or FDA-cleared.

### Carbapenemase detection using disk diffusion-based CA-SFM algorithm

The CA-SFM algorithm considers the inhibition diameters of ceftazidime-avibactam (<10 mm), temocillin (<16 mm), and meropenem or imipenem (<22 mm) (12). Inhibition zone diameters were obtained using the DD method (SirScan Disks, i2a) on Mueller-Hinton agar plates (Bio-Rad) incubated for 18 h at 35°C, according to EUCAST guidelines (http://www.eucast.org/).

### Data analysis

**Fig 1:**
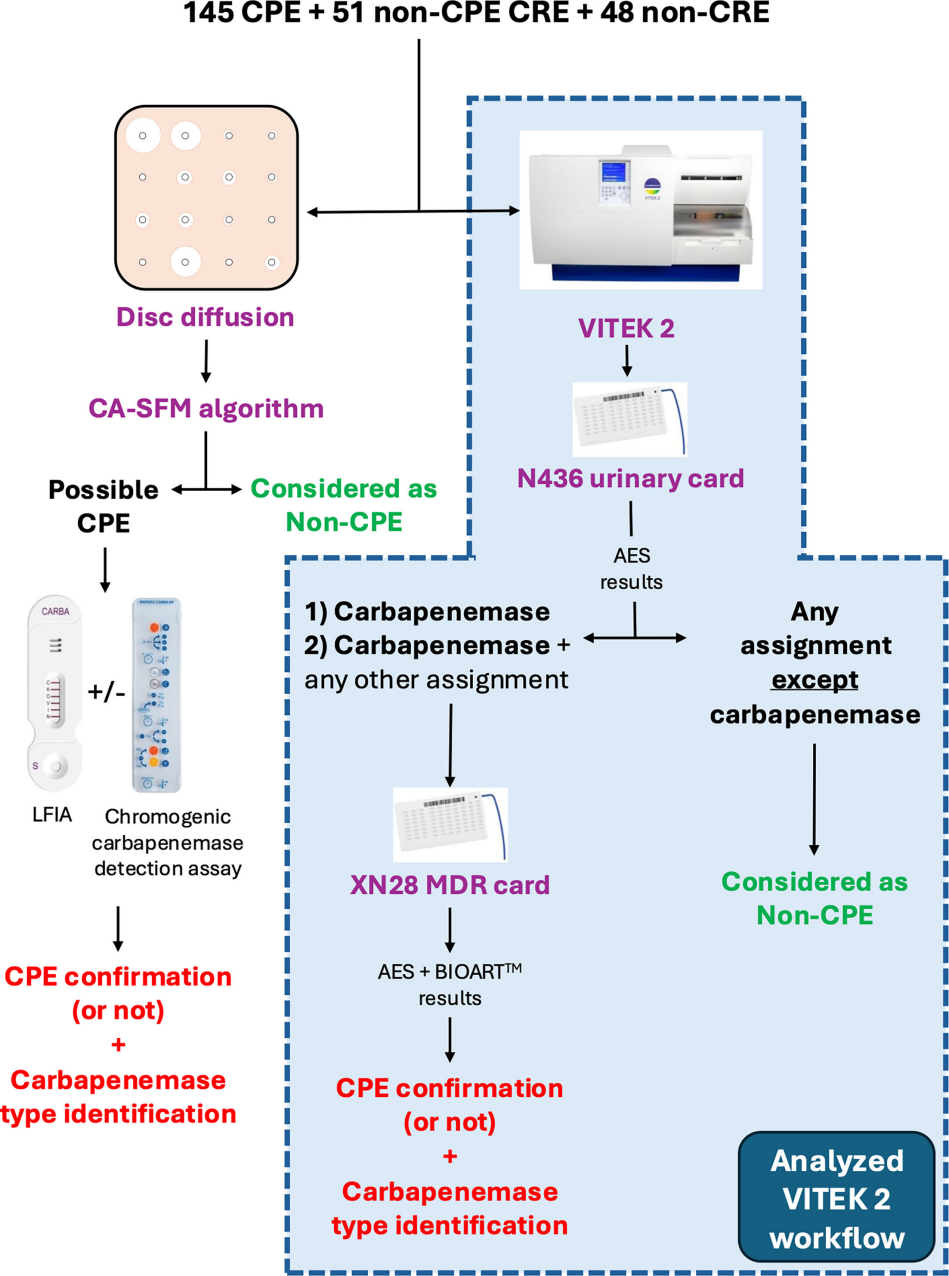
Scheme of the VITEK2 workflow used to interpret results in comparison with disk diffusion CA-SFM algorithm.

First, the performance of carbapenemase detection was evaluated for three screening approaches: the VITEK2 N436/AES card alone, the combined N436 + XN28/AES + BIOART rules approach (VITEK2 AES/BIOART solution), and the CA-SFM disk diffusion algorithm (Fig. 1).

In a second step, the ability of the AES combined with BIOART rules to classify carbapenemase types was assessed using a sequential workflow reflecting routine laboratory practice. This workflow consisted of an initial screening step using the N436 card (urinary card) interpreted by the AES. Isolates not detected as a possible carbapenemase-producing at this stage were not further analyzed. Accordingly, these isolates were considered as non-carbapenemase producers. For isolates detected as potential carbapenemase producers by N436/AES, a second step was performed using the XN28 card, triggering activation of the BIOART expert rules for carbapenemase class assignment (Fig. 1).

Sensitivities and specificities with 95% confidence intervals (95% CIs) were calculated. Comparisons of sensitivity and specificity between screening methods were performed using McNemar’s test. All statistical analyses were conducted using Stata software (version 17.0 SE, StataCorp LLC, College Station, TX, USA). For all analyses, a *P* value < 0.05 was considered statistically significant.

## RESULTS

### Characterization of the bacterial collection

A total of 244 isolates were included in the analysis, distributed as follows: *K. pneumoniae* (*n* = 80, 32.8%), *E. coli* (*n* = 79, 32.4%), *E. cloacae complex* (*n* = 45, 18.4%), *C. freundii* (*n* = 21, 8.6%), *K. oxytoca* (*n* = 7, 2.9%), *K. aerogenes* (*n* = 7, 2.9%), *S. marcescens* (*n* = 3, 1.2%), and *K. variicola* (*n* = 2, 0.8%).

Among the selected CPE isolates (*n* = 145), OXA-48-like enzymes were predominant (*n* = 71, 49%), and included a majority of OXA-48 (*n* = 39/71, 54.9%), followed by OXA-244 (*n* = 10/71, 14.1%), OXA-181 (*n* = 8/71, 11.3%), OXA-484 (*n* = 8/71, 11.3%), OXA-232 (*n* = 4/71, 5.6%), and OXA-204 (*n* = 2/71, 2.8%). MBL producers accounted for 37.9% of CPE, including NDM enzyme as the most frequent (*n* = 40/55, 72.7%), followed by VIM (*n* = 14/55, 25.4%) and IMP (*n* = 1, 1.8%). KPC and IMI enzymes were also identified, representing 4.1% (*n* = 6) and 1.4% (*n* = 2) of CPE, respectively. Finally, 11 isolates produced several carbapenemases, including 6 NDM + OXA-48-like, 2 OXA-48-like + VIM, 2 KPC + VIM, and 1 IMP + NDM.

Among non-CPE CRE isolates, CTX-M-type ESBLs (33.3%, 17/51) and high-level intrinsic AmpC producers (19.6%, 10/51) were the most prevalent. Finally, in the non-CRE group, most isolates produced ESBLs (89.6%, 43/48), with a minority expressing high-level *AmpC* (10.4%, 5/48) (Table S1).

### Detection of carbapenemase production

The sensitivity of CPE detection using the VITEK2 N436 urinary card alone and the CA-SFM algorithm reached 93.8% (136/145) and 97.2% (141/145), respectively, with no statisticalsignificance (*P* = 0.096) (Table 1). Adding the VITEK2 XN28 card did not significantly improve sensitivity (95.2%, 138/145, *P* = 0.27); specificities were 49.5% (49/99), 53.5% (53/99), and 52.5% (52/99) for the CA-SFM algorithm, VITEK2 N436 card, and VITEK2 N436 + XN28 (VITEK2 AES/BIOART solution), respectively, with no statistically significant difference across methods. Focusing on non-CRE isolates, specificities were 100% (48/48), 97.9% (47/48) and 95.8% (46/48) for CA-SFM algorithm, VITEK2 N436, and VITEK2 AES/BIOART solution, respectively (Table 1). The non-CRE isolates incorrectly detected as carbapenemase producers by the VITEK2 AES/BIOART solution corresponded to *E. cloacae* complex isolates co-producing ESBL and high-level intrinsic AmpC, showing borderline susceptibility to ertapenem.

**TABLE 1:** Performance of the VITEK2 N436 card, VITEK2 AES/BIOART solution, and CA-SFM algorithm for the detection of CPE^*a*^

				Number of isolates detected as CPE (%)		
	Resistance mechanism		*N*	CA-SFM algorithm	VITEK2 N436	VITEK2 AES/BIOART
CPE			145	141	136	138
	IMI		2	2 (100)	2 (100)	2 (100)
	KPC		6	5 (83.3)	6 (100)	6 (100)
	MBL		55	53 (96.4)	52 (94.5)	53 (96.4)
		NDM	40	39 (97.5)	40 (100)	40 (100)
		VIM	14	13 (92.9)	11 (78.6)	12 (85.7)
		IMP	1	1 (100)	1 (100)	1 (100)
	OXA-48-like		71	70 (98.6)	66 (93.0)	66 (93.0)
		OXA-244	10	9 (90.0)	6 (60.0)	6 (60)
		OXA-484	8	8 (100)	7 (87.5)	7 (87.5)
		Other OXA-48-like	53	53 (100)	53 (100)	53 (100)
	NDM + OXA-48-like		6	6 (100)	6 (100)	6 (100)
	NDM + KPC		2	2 (100)	2 (100)	2 (100)
	NDM + IMP		1	1 (100)	0 (0)	1 (100)
	VIM + OXA-48-like		2	2 (100)	2 (100)	2 (100)
Non-CPE			99	50	46	47
	CRE		51	50 (98.0)	45 (88.2)	45 (88.2)
	non-CRE		48	0 (0)	1 (2.1)	2 (4.2)
		% sensitivity (95% CI)		97.2 (93.1–99.2)	93.8 (88.5–97.1)	95.2 (90.3–98.0)
		*P* value		ref	0.096	0.27
		% specificity (95% CI)		49.5 (39.3–59.7)	53.5 (43.2–63.6)	52.5 (42.2–62.7)
		*P* value		ref	0.16	0.32

^*a*^ CPE, carbapenemase-producing Enterobacterales; CRE, carbapenem-resistant Enterobacterales.

When analyzed by CPE classes, the two IMI-producing isolates were detected as CPE phenotype with all tested methods. One KPC-producing strain was missed by the CA-SFM algorithm. The ability to detect MBL-producing strains was comparable between the CA-SFM algorithm, the VITEK2 N436, and the VITEK2 AES/BIOART solution. CA-SFM algorithm failed to detect one NDM-producer and one VIM-1 producer, whereas VITEK2 N436 missed three VIM-1 producers. Among OXA-48-like producers, CA-SFM algorithm missed one OXA-244-producing *E. coli* only, while five were not detected by the VITEK2 N436 and the VITEK2 AES/BIOART™ solution (including four OXA-244-producing *E. coli* and one OXA-484-producing *E. coli*) (Table 1). Interestingly, 3/5 of these isolates exhibited ertapenem MIC_VITEK_ of 0.25 mg/L using the VITEK2 N436. Accordingly, these OXA-244/-484-producing *E. coli* isolates would be detected as potential carbapenemase producers using the specific OXA-244/484 rule. All results obtained with both cards are detailed in Table S2.

### Determination of carbapenemase type

Since the VITEK2 N436 card demonstrated comparable performance to VITEK2 N436 + XN28 for carbapenemase detection, CPE type determination workflow was conducted sequentially, using card N436 as an initial screen for the potential presence of CPE (Fig. 1). Among the 145 CPE isolates, 136 (93.8%) were detected as carbapenemase-producing by the N436 card and were therefore eligible for carbapenemase class assignment with XN28 card (Table S2).

Among these 136 isolates, BIOART rules correctly identified the single carbapenemase class present with no other mechanism reported in 83 cases. In 30 cases, the correct carbapenemase class was reported together with additional interpretations, which could include either another carbapenemase class or non-enzymatic resistance mechanisms such as decreased membrane permeability. In six cases, no carbapenemase class was assigned despite detection of a carbapenemase phenotype (untyped carbapenemase), and in five cases an incorrect carbapenemase class was reported (Table 2; Table S2). Twelve isolates could not be evaluated for carbapenemase classification accuracy because they were outside the scope of BIOART expert rule assignments. These included two IMI-producing isolates and ten isolates producing multiple carbapenemases. The two IMI-producing isolates were reported as “untyped carbapenemase” by the BIOART rules. Because BIOART rules were not designed to detect multiple carbapenemase production, 9/10 MBL (NDM or VIM) + OXA-48-like or KPC co-producing isolates were reported as MBL producers.

**TABLE 2:** Results of carbapenemase detection and class determination using the sequential VITEK2 N436 and XN28 cards^*a*^

Carbapenemase type (reference method)	No. of isolates	AES phenotype categories (AST-N436)	False-negative results of AST-N436	False-positive results of AST-N436	No. of isolates tested with XN28	Combined AES + BIOART results (AST-N436 + XN28)	Correct class assignment (single class)	Correct but ambiguous class assignment	No class assignment	Incorrect class assignment
NDM	40	7 Carbapenemase (ESBL ±)	0% (0/40)	NA	40	39 MBL	97.5% (39/40)	0% (0/40)	0% (0/40)	2.5% (1/40)
		33 Carbapenem impermeability (ESBL ± or Hcase +) or carbapenemase (ESBL ±)				1 Carbapenem impermeability or OXA-48-like or KPC				
VIM	14	8 Carbapenem impermeability (ESBL ± or Hcase +) or carbapenemase (ESBL ±)	21.4% (3/14)	NA	11	8 MBL	57.1% (8/14)	0% (0/40)	14.3% (2/14)	7.1% (1/14)
		3 Carbapenemase (ESBL ±)				2 Untyped carbapenemase				
		2 Carbapenem impermeability (ESBL ± or Hcase +)				1 OXA-48-like				
		1 Non-concordant (ESBL or HL Case)								
IMP	1	1 Carbapenemase (ESBL ±)	0% (0/1)	NA	1	1 Untyped carbapenemase	0% (0/1)	0% (0/1)	100% (1/1)	0% (0/1)
OXA-48-like	71	40 Carbapenemase (ESBL ±)	7.0% (5/71)	NA	66	36 OXA-48-like	50.7% (36/71)	33.8% (24/71)	4.2% (3/71)	4.2% (3/71)
		26 Carbapenem impermeability (ESBL ± or Hcase +) or carbapenemase (ESBL ±)				15 Carbapenem impermeability or OXA-48-like				
		2 ESBL or Hcase				6 Carbapenem impermeability or OXA-48-like or KPC				
		2 Inhibitor-resistant penicillinase (IRT or OXA) or acquired penicillinase				3 Untyped carbapenemase				
		1 ESBL				3 OXA-48-like or KPC				
						2 Carbapenem impermeability or KPC				
						1 MBL				
KPC	6	6 Carbapenem impermeability (ESBL ± or Hcase +) or carbapenemase (ESBL ±)	0% (0/6)	NA	6	6 Carbapenem impermeability or KPC	0% (0/6)	100% (6/6)	0% (0/6)	0% (0/6)
IMI	2	2 Carbapenemase (ESBL ±)	0% (0/2)	NA	2	2 Untyped carbapenemase	NA	NA	NA	NA
NDM + OXA-48-like	6	5 Carbapenem impermeability (ESBL ± or Hcase +) or carbapenemase (ESBL ±)	0% (0/6)	NA	6	5 MBL	NA	NA	NA	NA
		1 Carbapenemase (ESBL ±)				1 Untyped carbapenemase				
OXA-48-like + VIM	2	2 Carbapenemase (ESBL ±)	0% (0/2)	NA	2	2 MBL	NA	NA	NA	NA
IMP + NDM	1	1 Carbapenem impermeability (ESBL ± or Hcase +)	100% (1/1)	NA	0		NA	NA	NA	NA
KPC + NDM	2	2 Carbapenemase (ESBL ±)	0% (0/2)	NA	2	2 MBL	NA	NA	NA	NA
Non-CP CRE	51	30 carbapenem impermeability (ESBL ± or Hcase +) or carbapenemase (ESBL ±)	NA	29.4% (15/51)	15	8 Untyped carbapenemase	NA	NA	NA	NA
		15 Carbapenemase (ESBL ±)				6 OXA-48-like				
		3 Hcase or ESBL				1 MBL				
		1 ESBL + Hcase								
		1 ESBL								
		1 Non-concordant (β-lactams: carbapenem impermeability (ESBL ± or HCase +), carbapenemase (ESBL ±))								
			
Non-CRE ESBL	43	36 ESBL	NA	0% (0/48)	0	NA	NA	NA	NA	NA
		3 Carbapenem impermeability (ESBL ± or Hcase +)								
		1 Hacse, ESBL								
		1 Acquired cephalosporinase (except ACC-1)								
		1 Carbapenem impermeability (ESBL ± or Hcase +)								
		1 Non-concordant								
Non-CRE Hcase	5	3 Hcase	NA	0% (0/5)	0	NA	NA	NA	NA	NA
		2 ESBL, Hcase								

^*a*^ CPE, carbapenemase-producing Enterobacterales; CRE, carbapenem-resistant Enterobacterales; Hcase, high level AmpC; NA, not applicable.

For the 66 OXA-48-like producers tested, the VITEK2 AES/BIOART solution yielded a fully correct and unique classification in 36 cases (54.5%), while 24 (36.3%) were correctly classified but with additional proposed mechanisms, mainly “carbapenem impermeability” (*n* = 15) (Table 2). Three isolates remained “untyped carbapenemase” and three isolates were misclassified. Among the 40 NDM producers, all were correctly identified as MBL producers except one isolate reported as carbapenem impermeability or carbapenemase (OXA-48-like) or carbapenemase (KPC) (Table S2). For the 14 VIM-producing isolates, 8 (57.1%) were correctly classified as MBL producers (Table S2). All 6 KPC-producing isolates were correctly recognized as KPC producers, but additional interpretations were systematically reported (possibility of carbapenem impermeability). The unique IMP-producing isolate of our collection was not recognized as MBL producer but reported as an untypeable carbapenemase (Table 2; Table S2).

### Validation of VITEK2 BIOART OXA-244 rule

Among the 71 OXA-48-like producers, 4/10 OXA-244 and 1/8 OXA-484 producers were missed as possible CPE using the N436 urinary card. As a result of the high false negative rate, a specific rule targeting OXA-244/OXA-484 variants was developed for *E. coli* isolates with an ertapenem MIC_VITEK_ of 0.25 mg/L. This additional VITEK2 OXA-244 BIOART rule was subsequently tested using a second set of 36 OXA-244/-484-producers (Table S3). The sensitivity for CPE phenotype detection using the first-line VITEK2 N436 card (without applying the dedicated OXA-244 rule) reached 94.4% (34/36). When applying the specific OXA-244 rule, all isolates were correctly detected as potential CPE. In comparison, the CA-SFM algorithm showed a lower sensitivity of 83.3% (30/36). After the addition of the XN28 card activating BIOART rules, the OXA-48-like class was proposed in 80.5% (29/36) of cases (20/36 with only OXA-48-like carbapenemase assigned, 1/36 with both OXA-48-like and KPC assigned, 7/36 with OXA-48-like and KPC and carbapenem impermeability assigned, and 1/36 with OXA-48-like and carbapenem impermeability assigned) (Table S3).

## DISCUSSION

The main objective of the study was to evaluate the performance of the VITEK2 AES combined with the BIOART rules, so-called VITEK2 AES/BIOART solution, for the detection and classification of CPE using a collection representative of the current French epidemiology. This collection was dominated by OXA-48-like carbapenemase producers, including several isolates producing OXA-244/OXA-484 enzymes, which are of growing concern in Europe (9, 10, 17).

The first line VITEK2 N436 card showed high sensitivity for detecting carbapenemase production, comparable to that of the CA-SFM algorithm (93.8% vs 97.2%). However, both methods showed low specificity, largely due to false-positive results among non-CPE CRE isolates with elevated carbapenem MICs (53.5% vs 49.5%). Specificity remained high for non-CRE strains (98.2% vs 100%). The CA-SFM algorithm performance was comparable to that reported in previous studies (97.8% and 45.5%) (11, 13). A recent study reported a superiority of CA-SFM markers algorithm adapted to VITEK2 (e.g., ceftazidime-avibactam, and/or temocillin, and/or carbapenems [imipenem or meropenem]) over an approach relying solely on a MIC_VITEK_ threshold >0.12 mg/L for at least one carbapenem, without using the phenotype provided by the AES (18). Importantly, this evaluation was performed using the older urine cards (N233 + XN12), which included the previous version of the ertapenem (etp01), and was conducted on rectal swabs and blood cultures isolates (18). In contrast, our analysis demonstrates similar performance between N436 with AES and CA-SFM algorithm when applying the updated ertapenem version (etp02) integrated in the new N436 urine card. The nine isolates not detected by the N436 card were mainly producers of carbapenemases with low hydrolytic activity, including four OXA-244 and one OXA-484 OXA-48-like variants, as well as three VIM-1 producers, whose hydrolytic activity is known to be lower than that of NDM enzymes. More unexpectedly, one *E. cloacae* complex isolate co-producing IMP and NDM was also not detected. Importantly, this study enabled the design and the evaluation of a dedicated OXA-244/484 BIOART rule aimed at detecting all OXA-244- and OXA-484-producing isolates, which were not identified as carbapenemase producers in the first round of experiments (5/18). Accordingly, the analysis of an independent collection of 36 OXA-244/-484-producing isolates demonstrated that the N436 card, when combined with this new OXA-244/484 BIOART rule, was able to detect 36/36 isolates. The lower detection rate observed in the main collection (72.2%, 13/18) compared with the extended OXA-244/-484 collection is likely related to the high prevalence of ST69 *E. coli* in the main data set, a clone exhibiting lower ertapenem MIC values leading to even more challenging detection.

Given that CPE are mainly isolated from urinary tract specimens among clinical samples (19), these findings support the use of N436 urinary card, combined with AES interpretation, as an appropriate first-line screening tool in routine laboratory settings before performing complementary tests (lateral flow immunochromatographic assays, chromogenic test for carbapenemase detection, or an additional VITEK2 card able to identify the carbapenemase class and determine susceptibility to alternative antimicrobials) in case of carbapenemase suspicion (Fig. 1). Importantly, the phenotype predicted by the VITEK2 AES is automatically incorporated into the MIC_VITEK_ report, facilitating the interpretation.

Using both cards, N436 + XN28 provide screening performances comparable to the N436 alone while delivering, in a single analytical step, both the full AST and the predicted carbapenemase type. The choice between a sequential workflow and a combined-card strategy (N436 + XN28 for all isolates) depends on laboratory resources, turnaround time constraints, and local epidemiology. In the present study, we opted to follow the routine laboratory workflow, which relies on sequential screening (Fig. 1). The XN28 was run for 93.8% of CPE isolates, and the performance reached in differentiating carbapenemase classes differed slightly from that reported by Eickhoff et al. in the context of US epidemiology (15).

The BIOART expert rules showed very good performance for the identification of MBL producers, particularly NDM-producing isolates, for which 97.5% were correctly assigned to the MBL class with a single and unambiguous proposition. Performances were also high for KPC-producers despite the carbapenemase class assignment being frequently affected by ambiguity, with decreased membrane permeability systematically co-proposed. For OXA-48-like producers, the algorithm proposed the correct class in 85% of cases, but a unique carbapenemase class was assigned in only 50% of isolates, reflecting frequent co-reporting of alternative mechanisms. It should be noted that no specific rule was defined for IMI, resulting in the absence of identification for this type of carbapenemase class. The archetypal phenotypic profile of IMI producers, characterized by high-level resistance to carbapenems without hydrolysis of extended-spectrum cephalosporins or aztreonam (20), allows relatively easy phenotypic identification and could be used to further improve BIOART expert rules. In addition, BIOART expert rules were not designed to identify the production of multiple carbapenemases, which constitutes an inherent limitation. In our study, all 10 multiple carbapenemase producers carried an MBL, among which nine were reported as MBL producers. From a clinical standpoint, this limitation is unlikely to have a major impact, as treatments commonly used for MBL-producing Enterobacterales, such as aztreonam-avibactam or cefiderocol, would also be effective against the multiple carbapenemase producers.

This study has several limitations that should be acknowledged. First, the performance evaluation was conducted on a strain collection representative of the French epidemiological landscape, which may not reflect the diversity and resistance profiles observed in other countries. In addition, the samples included a large number of CRE with high levels of resistance, resulting in the inability to calculate PPV and NPV. Finally, the CA-SFM approach used in this study did not incorporate Mueller-Hinton agar supplemented with cloxacillin, which is recommended for suppressing inducible intrinsic AmpC activity for group 3 Enterobacterales species (12, 21). This omission may have impacted the accuracy of phenotypic detection for some isolates, particularly those with borderline susceptibility profiles (21).

Overall, these findings indicate that the VITEK2 AES/BIOART solution offers a simple and rapid approach for CPE screening and classification while simultaneously providing MIC_VITEK_ data for a broad range of antibiotics. This integrated solution can support therapeutic decision-making and reduce the need for additional confirmatory tests, thereby lowering associated costs. Depending on the laboratory workflow, the two cards can be used either sequentially or in combination. Future improvements should aim to integrate carbapenemase class identification directly within the AES to simplify the results interpretation. Continued real-world evaluation is critical for adapting the system to local epidemiology and for assessing its clinical and economic impact.

## SUPPLEMENTAL MATERIAL

Table S1 jcm.00222-26-s0001.xlsx Resistance mechanism distribution among the tested samples.

Table S2 jcm.00222-26-s0002.xlsx Characterization of the 244 Enterobacterales isolates (CPE, non-CPE CRE, and non-CRE) included in this study, and corresponding results obtained using the VITEK 2 N436 card alone or in combination with the XN28 card (BIOART{trade mark, serif} rules). Color codes indicate the accuracy of carbapenemase class determination: red, discordant result; orange, correct carbapenemase class proposed among multiple interpretations; green, correct carbapenemase class proposed as a unique result.

Table S3 jcm.00222-26-s0003.xlsx Characterization of the 36 isolates producing OXA-244 or OXA-484 included in the complementary analysis, and corresponding results obtained using the VITEK 2 N436 card alone or in combination with the XN28 card (BIOART{trade mark, serif} rules). Color codes indicate the accuracy of carbapenemase class determination: red, discordant result; orange, correct carbapenemase class proposed among multiple interpretations; green, correct carbapenemase class proposed as a unique result.

Table S4 jcm.00222-26-s0004.xlsx Antibiotic composition of the VITEK 2 N436 and VITEK 2 XN28 cards.

ASM does not own the copyrights to Supplemental Material that may be linked to, or accessed through, an article. The authors have granted ASM a non-exclusive, world-wide license to publish the Supplemental Material files. Please contact the corresponding author directly for reuse.
